# Comparison of two questionnaire for subjective symptoms of dry eye in patinents with schizophrenia

**DOI:** 10.1192/j.eurpsy.2022.436

**Published:** 2022-09-01

**Authors:** I. Bakija, I. Filipcic, M. Bogadi, S. Kaštelan

**Affiliations:** 1 Psychiatric Clinic “Sveti Ivan”, Psychiatry, Zagreb, Croatia; 2 Psychiatric Hospital for children and adolescents, Department For Adolescent Psychiatry, Zagreb, Croatia; 3 Clinical Hospital Dubrava, Department Of Ophthalmology, Zagreb, Croatia

**Keywords:** dry eye disease - Schein questionnaire - OSDI questionnaire - schizophrenia

## Abstract

**Introduction:**

Psychiatric disorders may be one of considerable contributing factors for dry eye symptoms and the severity of subjective symptoms of dry eye are often related to psychological factors.

**Objectives:**

The aim of this research was to determine which of two chosen questionnaires for subjective symptoms of dry eye (Ocular Surface Disease Index and Schein questionnaire) is more reliable in the assessment of dry eye in patients with schizophrenia.

**Methods:**

Our research included 80 patients of both sexes with schizophrenia ranging between the age of 25 and 55 who have been taking antipsychotics (clozapin, olanzapin, quetiapin) for five years and were in remission. All participants were required to satisfy all included and excluded criteria. They all filled out the Schein and OSDI questionnaires for assessment of subjective symptoms. Tear break-up time test (TBUT) for objective evaluation of tear film stability was also performed. In order to determine the correlation between two subjective and objective tests we calculated Spearmans correlation coefficients.

**Results:**

A analysis shows that there are no statistically significant differences between the correlations. Both subjective questionnaires are statistically significantly and negatively related to the TBUT test, showing that an increase in the results on the OSDI and Schein’s questionnaires led to the decreases in the results on the TBUT test.

**Conclusions:**

In patients with schizophrenia the OSDI and Schein questionnaires are equally reliable in the assessment of subjective symptoms of Dry eye disease. Considering that, OSDI is more common in clinical practice, it is recommended for use in patients with schizophrenia.

**Disclosure:**

No significant relationships.

